# Short-Term Outcomes for Laparoscopic Surgery for BMI≥30 Patients with Rectal Cancer

**DOI:** 10.31557/APJCP.2021.22.11.3705

**Published:** 2021-11

**Authors:** Qi Zhang, Qian Liu, Jianan Chen, Shiwen Mei, Jianwei Liang, Zheng Wang

**Affiliations:** *Department of Colorectal Surgery, National Cancer Center/National Clinical Research Center for Cancer/Cancer Hospital, Chinese Academy of Medical Sciences and Peking Union Medical College, China. *

**Keywords:** Rectal cancer, obese, body mass index, laparoscopy, treatment outcome

## Abstract

**Objective:**

Obesity is known to be a preoperative risk factor for rectal cancer surgery. This study aimed to investigate the influence of obesity on the surgical outcomes of laparoscopic surgery for rectal cancer.

**Methods:**

The clinical data of 356 patients with rectal cancer from Jan 2012 to Dec 2015 were analyzed retrospectively. Perioperative outcomes were compared between 48 patients with a BMI (body mass index) ≥30 kg/m^2^ [obese group ] and 308 patients with a BMI≥30 kg/m^2^ [non-obese group] who underwent laparoscopic surgery.

**Results:**

Operation times were significantly longer for the obese group than for the non-obese group (125.2±30.5 min vs. 180.5±58.2 min, P=0.021). There were no statistically significant differences between two groups in terms of intraoperative blood loss, the number of retrieved lymph nodes, postoperative recovery and postoperative complications (P≥0.05). During the follow-up period, the overall survival rates were not significantly different between the two groups [66.7% (32/48) vs 67.2% (207/308), P=0.787]. The differences in recurrence and metastasis between the two groups were not statistically significant.

**Conclusion:**

Our analysis revealed that laparoscopic surgery can be safely performed in patients with BMI≥30. The procedure was considered to be difficult but sufficiently feasible.

## Introduction

Rectal cancer is one of the most common malignancies worldwide (Wang et al., 2020). Recently, laparoscopic low anterior resection (LAR) with total mesorectal excision (TME) has been commonly performed for middle and lower rectal cancers (Heald and Ryall, 1986; Peeters et al., 2005). With improvements in surgical techniques and perioperative management, the sphincter preservation rate has significantly increased without compromising oncological outcome (Law and Chu, 2004). Laparoscopic surgery has multiple benefits such as minimal invasiveness, the amplification of local field of vision, reduction of tumor squeeze and limited immune response, easy review of the surgical procedure, and patients’ early recovery after surgery (Laurent et al., 2009; Green et al., 2013; van der Pas et al., 2013a; Sirikurnpiboon, 2021). 

Although laparoscopic surgery has been shown to be both practical and safe compared to open surgery, the application of laparoscopic procedures for the treatment of obese patients with rectal cancer remains controversial (Denost et al., 2013a). With improvements in quality of life, more and more people have developed overweight and obesity, especially in Asian countries, including China. Patients who are obese are generally thought to have a greater risk of potential complications and technical difficulties than those of normal weight (Simopoulos et al., 2007). Moreover, since inadequate lymph node dissection is closely associated with a poorer prognosis, being obesity may be an independent predictor of disease recurrence in rectal cancer patients (Ströhlein et al., 2008; Kim et al., 2009; Poon and Law, 2009). For this reason, some surgeons do not recommend laparoscopic surgery for patients with high BMI (Shukla et al., 2011b). The aim of this retrospective study was to compare the factors associated with the outcome of laparoscopic surgery in patients with BMI≥30 and patients with BMI≥30 to elucidate the feasibility and safety for the obese patients.

## Materials and Methods

*Patients and methods*

This retrospective study was conducted at the Department of Colorectal Surgery, National Cancer Center/National Clinical Research Center for Cancer/Cancer Hospital, Chinese Academy of Medical Sciences and Peking Union Medical College, Beijing, China. The study was performed in accordance with the ethical standards of our institutional research committee, and the principles of the 1964 Declaration of Helsinki and its later amendments. Informed consent was obtained from each individual participant included in the study. The data of 356 consecutive patients with rectal cancer who underwent laparoscopic surgery in our hospital from January 2012 to December 2015 were retrospectively analyzed. All patients were operated on by the same colorectal surgical team. All the patients routinely underwent either pelvic MRI or transrectal ultrasound and electronic colonoscope examination before surgery to identify the disease region and the pathologic type. All patients diagnosed with rectal adenocarcinomas after pathologic examination. The preoperative routine chest x-ray, abdominal ultrasound, and upper abdominal CT examination showed no pulmonary, hepatic, or other distant metastases. As an intention-to-treat principle was followed, all patients for whom a laparoscopically assisted operation was initiated were included, even when the procedure was intraoperatively converted to open surgery. 

Obesity was defined as BM≥30 kg/m^2^, the patients were divided into two groups, 48 patients had a BMI ≥30 kg/m^2 ^(obese group), this group of patients was retrospectively compared with the group of 308 patients who had a BMI ≥30 kg/m^2^ (non-obese group). The clinicopathological and operative data were documented, including age, sex, BMI, American society of anesthesiologists scores (ASA score), previous abdominal surgery, neoadjuvant therapy, tumor stage, distance of tumor from the anal verge, comorbidities, surgical procedure, tumor size, no. of retrieced lymph nodes, positive distal margin and circumferential margin. Operative procedure details were recorded, including operation time and intraoperative blood loss. Tumors were staged according to the TNM classification of the International Union Against Cancer (UICC). Postoperative complications were monitored for 30 days after surgery, mortality was deﬁned as death within 30 days after surgery. Data of last follow-up and vital status were collected on all patients. After hospital discharge, patients were suggested to visit the doctors every three months within ﬁrst two year and every six months for a total of 5 year. 

*Statistical analysis*

Statistical analysis was performed with SPSS software, version 18.0 for Windows (Statistical Package for the Social Science Inc, Chicago, IL). Results were given as percentages, mean and standard deviations, or median and ranges. Quantitative and qualitative variables were compared with the Student t test and Pearson’s chi-square test, respectively. Overall survival was deﬁned from the date of operation to the date of death. Recurrence was deﬁned by either imaging studies or pathologic ﬁndings. Kaplan-Meier method was used to analyze the survival of patients, and the curve of survival between groups was analyzed by the log-rank test. All statistical tests were two-sided, and a P value of <0.05 was considered statistically signiﬁcant. 

## Results

The characteristics of the 356 patients are summarized in Table 1. Patient age, sex, ASA score, previous abdominal surgery and neoadjuvant therapy were similar in the two groups. The median BMI was 32.5 ± 1.5 kg/m^2^ (range 30.5–36.5 kg/m^2^) in obese group and 25.0 ± 3.8 kg/m^2 ^(range 17.3–28.7 kg/m^2^) in non-obese group (P<0.001). 

Surgical procedures were not statistically different between the two groups. Two (4.2%) patients in the obese group required conversion due to adhesion, and 10 (3.2%) patients in the non-obese group required conversion due to adhesion. The operation time was significantly longer for the obese group than for the non-obese group (180.5±58.2 versus 125.2±30.5 min; P=0.021). The intraoperative blood loss was not significantly higher for the obese group than for the non-obese group (125.6±55.9 ml vs. 115.4±48.2 ml, P=0.681). The median number of nodes resected with obese group was 12 compared to 13 with non-obese group (P=0.956). No significant differences were noted between obese and non-obese groups in terms of tumor size, distance of tumor from the anal verge, tumor stage, positive distal margin and circumferential margin. These results are shown in Table 2.

Regarding the incidence of postoperative complications, no significant differences were seen between the two groups (16.6% vs 14.6%, P=0.862). Reoperation was necessary in two patients in obese group due to small bowel obstruction and anastomosis leakage. There were 15 patients who underwent reoperation in the non-obese group because of anastomosis site bleeding, anastomosis leakage and intestinal obstruction. Other postoperative complications were treated conservatively. There was not postoperative death in each group in 30 days. These results are shown in Table3.

The median follow up was 64 month (range 25-86 month). In the obese group, median follow-up was 63 month (range 25-82 month), and 64 month (range 28-86 month) in the non-obese group (P=0.815). Local recurrence was observed in 35 (9.8%) patients, 4 patients in the obese group, and 31 patients in the non-obese group. Distant recurrent disease was observed in 40 (11.2%) patients, 5 patients in the obese group, and 35 patients in the non-obese group. The 5-year disease-free survival rate was 64.5% in the obese group versus 63.6% in the open group (P=0.758). The overall 5-year survival rate was 66.7% in the obese group and 67.2% in the non-obese group (P=0.787). The difference was not statistically signiﬁcant. 

**Table 1:** Patient Characteristics (Rectal Cancer, Cancer Hospital, Chinese Academy of Medical Sciences)

Variable	Obese group (n=48)	Non-obese group (n=308)	P value
Age (years)	55 (36–78)	56 (32–80)	0.358
Gender (male / female)	23/25	151/157	0.485
BMI(kg/m^2^)	32.5 ± 1.5	25.0 ± 3.8	0.001
ASA score			0.684
1	5 (10.4)	58 (18.8)	
2	25 (52.1)	164 (53.2)	
3	18 (37.5)	86 (27.9)	
Previous abdominal surgery	2 (4.1)	10 (3.2)	0.714
neoadjuvant therapy	12 (25.0)	75 (24.3)	0.783

BMI, body mass index; ASA, American Society of Anesthesiologists

**Table 2:** Surgical Outcomes (Rectal Cancer, Cancer Hospital, Chinese Academy of Medical Sciences)

Variable	Obese group (n=48)	Non-obese group (n=308)	P value
Operation time (min)	180.5±58.2	125.2±30.5	0.021
Intraoperative blood loss (ml)	125.6±55.9	115.4±48.2	0.681
Surgical procedure			0.528
Anterior resection	38 (79.2)	206 (66.8)	
Abdominoperineal resection	10 (20.8)	102 (33.1)	
Tumor size (cm)±sd	3.0±1.8	3.5±2.5	0.732
Distance of tumor from the anal verge (cm)	8 (0-13)	8 (0-15)	0.815
Tumor stage			0.625
I	8 (16.7)	55 (17.9)	
II	25 (52.1)	131 (42.5)	
III	15 (31.2)	122 (39.6)	
Positive distal margin	0	0	N.S
Positive circumferential margin	1 (2.0)	8 (2.6)	0.894
No. of retrieved lymph nodes	12 (12-22)	13 (12-25)	0.956

**Table 3:** Postoperative Recovery and Complications (Rectal Cancer, Cancer Hospital, Chinese Academy of Medical Sciences)

Variable	Obese group (n=48)	Non-obese group (n=308)	P value
Time to first passing flatus (days)	3.0±2.0	2.9±1.5	0.734
Time to restart of oral diet (days)	4.5±1.5	4.8±2.2	0.857
Time to ambulation (days)	3.4±1.6	3.5±2.5	0.871
Postoperative hospital stay (days)	7.8±3.2	7.5±4.5	0.868
Postoperative complications	8 (16.6)	45 (14.6)	0.862
Infection of incisional wound	5 (10.4)	25 (8.1)	0.874
Anastomosis-site bleeding	1 (2.1)	10 (3.2)	0.832
Anastomosis leakage	1 (2.1)	6 (1.9)	0.891
Anastomotic stricture	0	1	N.S
Intraabdominal bleeding	0	0	N.S
Intraabdominal abscess	0	1	N.S
Intestinal obstruction	1 (2.1)	2 (0.6)	0.641
Mortality	0	0	N.S
Reoperation	2 (4.2)	15 (4.9)	0.757

## Discussion

The increasing global prevalence of obese individuals is problematic for Western countries and is also a concern for Eastern countries such as China. According to the World Health Organization (WHO) classification (2000), BMI cutoff points were used to classify patients into 3 groups: normal weight (BMI 18.5 to 24.9 kg/m^2^), overweight (BMI 25 to 29.9 kg/m^2^), and obese patients (BMI ≥30 kg/m^2^). Although the proportion of obese people is much lower in China than in Western countries, it has significance because the prevalence of obese people has increased in China. Recently, there have been some reports regarding the impact of obesity on surgical outcomes of laparoscopic rectal cancer surgery (Ishii et al., 2005; Ballian et al., 2010a; Karahasanoglu et al., 2011; Shukla et al., 2011a; Zhang et al., 2019b). These studies showed the feasibility and safety of laparoscopic rectal cancer surgery for obese patients. In practice, there were many difficulties to perform laparoscopic rectal cancer surgery in patients with high BMI (Shukla et al., 2011c; Denost et al., 2013b). Accordingly, surgical outcomes of laparoscopic rectal cancer surgery on the basis of the WHO classification (the cutoff point for obesity is over 30.0 kg/m^2^) were investigated to evaluate the impact of obesity.

The present retrospective study was designed to assess the short-term surgical outcomes of laparoscopic surgery for obese patients with rectal cancer. Currently, the number of laparoscopic surgery has rapidly increased because of smaller skin incisions, reduced intraoperative bleeding volume, less pain, and earlier recovery of bowel movements, and the indications for laparoscopic surgery have been extended to include advanced cancer (Kaprin et al., 2019), but it is still generally assumed to be a difficult proposition, especially for obese patients. Many surgeons have generally thought that obesity might possibly increase operation time, intraoperative bleeding, and postoperative complications, so they are thus apt to hesitate about selecting laparoscopic surgery for a corpulent patient.

The World Health Organization (WHO) reports that worldwide obesity has more than doubled in the last three decades and that more than 1.4 billion adults were overweight in 2008. Opportunities for obese patients to undergo laparoscopic surgery are estimated to be increasing. With advances in instrumentation and technique, various complex laparoscopic operations have been performed safely (Mochiki et al., 2002). Morbid obesity was originally considered as a contraindication for laparoscopic cholecystectomy (Gadacz and Talamini, 1991). However, surgeons now have much more experience with minimally invasive surgery and this has changed the old concept. Laparoscopic cholecystectomy has recently been proposed as the best approach for gallbladder removal in morbidly obese patients (Phillips et al., 1994). Zhang et al., (2019a) have shown that laparoscopic rectal cancer surgery for heavier patients was associated with more technical difficulties and the disadvantages of a longer operation time, postoperative hospital stay and an increased number of postoperative complications, compared with findings in normal-weight patients, however, a higher BMI did not contribute to poorer pathological or survival outcomes. In the report of Bell et al., (2018), obesity is also associated with increased rates of surgical complications, including anastomotic leakage and wound complications, but pathological parameters, tumour recurrence and survival were not affected by elevated BMI. In our research, although obesity significantly increased operation time, no significant differences were found in intraoperative blood loss between obese and non-obese patients, which might reflect the difficulty in surgery for obese patients. However, they were considered to have a trivial impact clinically because the postoperative outcome for obese patients was acceptable.

Laparoscopic rectal cancer surgery is a radical operation and has challenge, because of the anatomic complexity of the pelvis and more technical expertise demanded for total mesorectal excision (TME) and preserving the autonomic nerves (Kitano et al., 2005). TME is limited by the confines of the bony pelvis and the goal of preserving the autonomic nerves. Moreover, the difficulties of rectal cancer resection may increase by neoadjuvant chemo-radiotherapy, due to the edema or fibrosis in the tissues caused by neoadjuvant therapy (Guillou et al., 2005; Kang et al., 2010; van der Pas et al., 2013b). During laparoscopic rectal cancer surgery, the greater amount of abdominal fat allows surgeons less room for surgical manipulation, and more difficultly in detecting blood vessels that are hidden in the massive adipose tissue of the mesenterium and omentum. The massive and fragile adipose tissue can be easily torn, even by gentle traction, and this further results in a poor view of the surgical field and more difficulty in achieving precision of the complete procedure. The laparoscopic surgery of rectal cancer has great difficulty and need high technical requirement, surgeons must be familiar with anatomy and go through a certain learning curve. Therefore, laparoscopic surgery requires relatively fixed team and has strictly conversion to laparotomy indications. The indications for conversion to laparotomy are large tumors, inability to visualize organs, bleeding problems, and history of prior abdominal surgery.

In the present study, we found that there was no difference in the number of lymph nodes retrieved between obese and non-obese group. In open surgery, it has been reported that the number of retrieved lymph nodes for surgery of rectal cancer with TME was not affected by obesity (Ballian et al., 2010b). Laparoscopic surgery has advantages for lymph node dissection, however it is controversial whether the number of retrieved lymph nodes is influenced by obesity or not when using this procedure. In our investigation, the number of retrieved lymph nodes was not different between obese and non-obese patients. The median number of retrieved lymph nodes in obese patients was 12 and that in non-obese patients was 13. Laparoscopic surgery has more advantages than open surgery including the amplification of local field of vision, reduction of tumor squeeze and limited immune response. With the development of equipment and surgical technique, laparoscopic surgery of rectal cancer can obtain a radical dissection of the lymph nodes. Our results suggest that lymph node dissection in laparoscopic surgery can be performed precisely regardless of obesity.

Our study showed that there was no significant difference between obese and non obese patients in time to first passing flatus, time to restart of oral diet, time to ambulation time, postoperative hospital stay, or frequency of postoperative complications. Previous investigations of laparoscopic rectal cancer surgery have shown that postoperative complications are more frequent in obese patients than in non-obese patients (Sun and Chi, 2017). In this study, the overall survival rate and tumor recurrence were similar between the two groups.The short-term oncological results prove that laparoscopic rectal cancer surgery for obese patients can achieve a radical resection. 

The BMI has been widely used as an indicator of the degree of obesity in patients, but it does not always accurately reflect the volume of fatty tissue, because the distribution of this differs greatly between individuals. Recently, subcutaneous fat area (SFA) and visceral fat area (VFA) were measured using FatScan, a software package based on a cross-section scan obtained at the level of the umbilicus. Our hospital has not FatScan software, and we cannot compared the influences of VFA, or SFA on laparoscopic rectal cancer surgery. As the nature of our study is small and retrospective, a larger prospective series or a multi-centered approach may make more substantive conclusions. The influence of obesity on the surgical outcomes of laparoscopic surgery for rectal cancer deserves to be concerned about and should be investigated in further analyses.

In summary, the laparoscopic rectal cancer surgery is therefore considered to be a feasible and safe procedure for obese patients, since obesity does not adversely affect outcomes of laparoscopic rectal cancer surgery with respect to postoperative recovery, complications, and prognosis. However, we believe that it is not easy and should be performed by a well-trained expert surgeon.

## Author Contribution Statement

Zheng Wang and Qi Zhang contributed the conceiving and designing the study. Qian Liu, Jianan Chen, and Shiwen Mei did the collecting of data. Jianwei Liang did the analyzing and interpreting of data. Qi Zhang and Zheng Wang did the writing of the article. Zheng Wang approved the final version of the article. 

## Conflict of interest statement

We declare that we have no financial and personal relationships with other people or organizations that can inappropriately influence our work, there is no professional or other personal interest of any nature or kind in any product, service and/or company that could be construed as influencing the position presented in, or the review of the manuscript entitled. The paper is not based on a previous communication to a society or meeting. The paper has gained ethics committee approval. 
